# Experimental Study on Methane Explosion Suppression in Pipelines by Mixed Systems of Inert Gases (CO_2_/N_2_) and HFC-227ea

**DOI:** 10.1021/acsomega.6c05799

**Published:** 2026-10-06

**Authors:** Zhie Wang, Haixia Zhang, Qingcheng Zhang, Junhai Liu, Jingde Xu, Qing Wang, Yalong Jiang, Xueli Liu

**Affiliations:** † Urban Construction College, 195373 Anhui Xinhua University, Hefei, Anhui 230088, China; ‡ School of Safety Engineering, 71039 North China Institute of Science and Technology, Langfang, Hebei 065201, China; § School of Science and Technology, 91594 Anhui University of Science and Technology, Huainan, Anhui 232001, China; ⊥ Key Laboratory of Building Structure of Anhui Higher Education Institutes, 195373 Anhui Xinhua University, Hefei, Anhui 230088, China

## Abstract

To address the high inert gas demand and cost of heptafluoropropane (HFC-227ea) in gas explosion prevention, a custom-built pipeline experimental platform was employed to investigate the suppression effects of CO_2_, N_2_, and HFC-227ea on 9.5% methane explosions. A BOPP film separated the explosion and suppression zones to simulate nonpremixed conditions. The peak overpressure and average flame propagation speed were analyzed to evaluate suppression characteristics and the influence of film-breaking. Results show that CO_2_ outperforms N_2_ at equal volume fractions; both achieve the optimal suppression effect within the tested concentration range at 40%, reducing the peak overpressure by 25.8% and 24.4% and the flame speed by 40.1% and 34.0%, respectively. HFC-227ea exhibits strong chemical inhibition at low concentrations (2–8%), achieving 51.4% flame speed reduction at 8%. In mixtures with the total concentration fixed at 40%, suppression performance improves with higher HFC-227ea proportion. The 32% CO_2_/8% HFC-227ea mixture reduces the peak overpressure by 35.3% and the flame speed by 62.9%, outperforming pure CO_2_ at the same total concentration. No marked synergy is observed at low HFC-227ea ratios, likely due to film-breaking effects induced by BOPP membrane rupture. These findings provide theoretical and technical support for optimizing explosion suppression media in confined industrial spaces, suggesting a potentially cost-effective solution for engineering applications.

## Graphical abstract

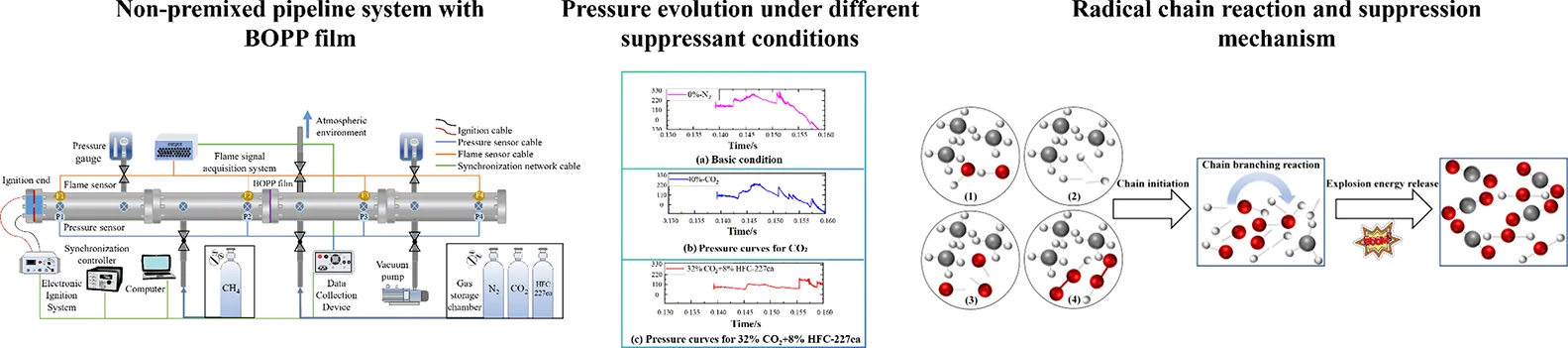

## 1 Introduction

In the context of the global low-carbon energy transition, coal remains a fundamental primary energy source and plays an irreplaceable role in ensuring a stable energy supply in both industrialized and developing countries.^1–3^ Methane explosion is among the most destructive hazards in underground coal mining worldwide, characterized by rapid detonation wave propagation, severe overpressure damage, pronounced chain effects in secondary disasters, and high casualty rates. It has long been a core bottleneck restricting the safe and sustainable development of the global coal industry.^4–6^ Statistics indicate that over the past decade, methane explosions have accounted for more than 50% of major coal mine accidents globally, not only resulting in significant casualties and substantial economic losses but also severely disrupting mine production operations worldwide.^7^ Consequently, the development of efficient, safe, and economical active methane explosion prevention and control technologies has remained a central scientific challenge and a major engineering demand in the fields of mine safety and combustion dynamics.^8,9^

Within the existing technical framework for methane explosion suppression, gaseous explosion suppressants have been widely adopted in global mining operations due to their advantages, such as strong diffusivity, the absence of dead zones in explosion suppression, and suitability for the complex underground roadway environment.^10^ Focusing on the explosion suppression characteristics of inert gases, scholars have conducted systematic investigations. Bundy et al.^11^ determined the critical concentrations of nitrogen (N_2_), carbon dioxide (CO_2_), and bromotrifluoromethane (CF_3_Br) required to completely suppress methane explosion flames, confirming the stable nature of these critical inhibition concentrations. Di Benedetto et al.^12^ observed through closed-vessel experiments that the explosion overpressure and pressure rise rate were negatively correlated with the CO_2_ concentration. Chemkin simulations revealed the underlying mechanism by which CO_2_ suppresses explosions through reductions in laminar burning velocity, flame temperature, and combustion rate. Senecal^13^ established a flame quenching model based on the optimal quenching concentration of inert gases. Wang et al.^14^ confirmed that the explosion suppression efficacy of N_2_ and CO_2_ increases with concentration, with CO_2_ exhibiting superior performance. Given the limitations of single inert gases, such as the need for high concentrations and limited suppression efficiency, multicomponent composite explosion suppression systems have become a research focus. Pei et al.^15^ found that the synergistic effect of N_2_ or CO_2_ combined with ultrafine water mist significantly reduced the maximum flame propagation velocity, peak temperature, and peak overpressure. Wang et al.^16^ demonstrated that the suppression effect of water mist containing KCl coupled with CO_2_ on shock wave overpressure and flame velocity outperforms that of coupling with N_2_. Li et al.^17^ investigated the synergistic explosion suppression effect of inert gases combined with ABC dry powder. They characterized the influence of the ignition delay time on the suppression efficacy of ultrafine ABC dry powder. With advancing research, HFC-227ea, as an efficient chemical explosion suppressant, has garnered increasing attention in the field of gas explosion suppression. Cao et al.^18^ found that HFC-227ea effectively reduces the explosion reaction rate, and its suppression effect is further enhanced when used in combination with inert gases. Yu et al.^19^ reported that 5% HFC-227ea alone can completely suppress an explosion, whereas 25% CO_2_ is required to achieve a comparable effect, revealing the core mechanism of HFC-227ea’s high efficiency in quenching chain reaction radicals. Ji et al.^20^ showed that modified ABC powder driven by HFC-227ea can form an aerosol that achieves full contact and reaction with the flame, and the combination of the two exhibits a strong synergistic explosion suppression effect.

Although HFC-227ea exhibits a remarkable methane explosion suppression effect at low concentrations, two core limitations persist in existing research, constraining the underground engineering application of the inert gas/HFC-227ea composite explosion suppression technology. First, most related studies adopt an experimental approach involving the premixing of the suppressant with methane, which deviates significantly from the actual coal mine condition where the suppressant and combustible gas are spatially separated. It thus fails to reflect the dynamic explosion suppression process in real-world engineering scenarios. Second, as a key factor influencing the release, mixing, and action effects of suppressants under nonpremixed conditions, the interference mechanism and quantitative impact of the film-breaking effect on the synergistic explosion suppression performance of mixed systems remain unclear. Consequently, systematic investigations into the explosion suppression characteristics, quantitative synergistic effects, and microscopic mechanisms of inert gas and HFC-227ea mixed systems under nonpremixed conditions are still lacking.

To address these research gaps, this study employs a horizontal pipeline explosion experimental system in which a BOPP film separates the explosion zone from the suppression zone, simulating the nonpremixed, spatially separated condition of suppressant and combustible gas in coal mines. The work systematically investigates the explosion suppression characteristics and synergistic mechanisms of inert gas/HFC-227ea mixed systems and reveals the influence of the film-breaking effect on the synergistic suppression performance of the mixed system. The specific research contents include the following:

(1) Suppression experiments using single-component suppressants, including CO_2_, N_2_, and HFC-227ea, under varying concentration gradients to establish the concentration–efficiency response relationship. We elucidate the mechanistic differences among physical dilution, thermal absorption, and chemical suppression and develop a benchmark database for single-component explosion suppression efficiency.
(2) With the total volume fraction of the suppressant fixed at 40%, we systematically vary the mixing ratio of inert gases and HFC-227ea to investigate the enhancement patterns and synergistic effects of the mixed system on the explosion suppression performance. Combined with pressure–time history curves and flame propagation characteristics, we analyze the interference mechanism of the film-breaking effect on the synergistic suppression of the mixed system and clarify the coupling regulation mechanism of physical dilution and chemical inhibition under nonpremixed conditions.

Through the above investigations, this work aims to determine the optimal explosion suppression ratio and synergistic behavior of the inert gas/HFC-227ea mixed system under nonpremixed conditions, thereby providing theoretical support and an experimental basis for the development of efficient, safe, and economical methane explosion suppression technologies for coal mines.

## 2 Methodology

### 2.1 Experimental System

This experiment employed a horizontal pipeline methane explosion suppression experimental system (as shown in Figure 1), which accurately simulates the conditions of methane explosion propagation and active explosion suppression in underground coal mine roadways. The system consists of four main components: the explosion pipeline assembly, the gas mixing and vacuum unit, the ignition and synchronization control unit, and the signal acquisition and optical observation units.

**Figure 1:**
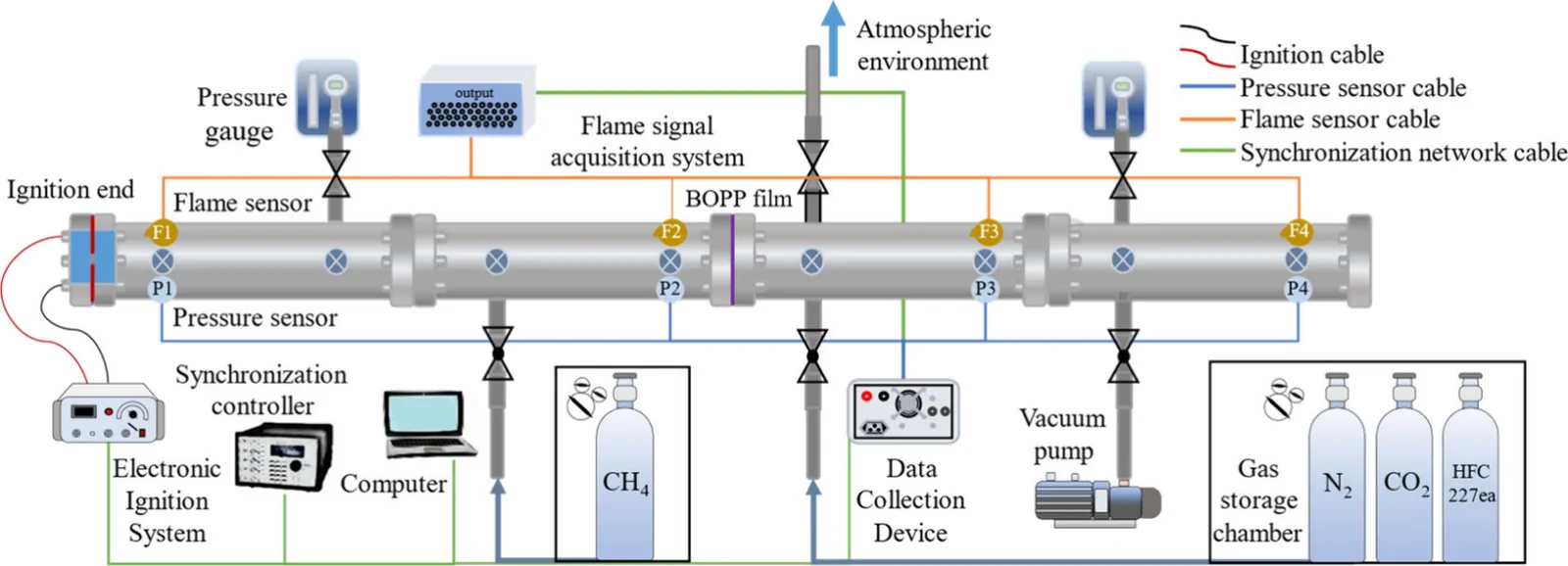
Schematic diagram of the experimental setup.

The explosion pipeline is made of 304 stainless steel with an inner diameter of 90 mm, a wall thickness of 10 mm, and a total length of 12 m. Taking the ignition end as the origin (*x* = 0 m), the pipeline is divided into an explosion zone (0–6 m) and a suppression zone (6–12 m) by a BOPP film installed at *x* = 6 m, which replicates the engineering scenario where the suppressant and combustible gas are spatially separated in underground roadways. Pairs of pressure sensors and flame photoelectric sensors are arranged along the pipeline axis at *x* = 0.5, 5.5, 8.5, and 11.5 m. The key monitoring point P3 analyzed in this study is located at *x* = 8.5 m, which is 2.5 m downstream of the BOPP film and lies within the stable flame propagation section after the film rupture disturbance. Observation windows are also set at corresponding positions to enable synchronous monitoring of the entire explosion process. The gas mixing system prepares the experimental gas mixture using Dalton’s law of partial pressures, with a relative concentration control error of ≤0.1%. The vacuum unit employs a rotary vane vacuum pump capable of evacuating the pipeline to a high vacuum, and a high-precision gauge monitors the internal pressure in real time. The ignition system is a high-voltage electric spark igniter with a fixed ignition energy of 20 mJ and an ignition voltage of 2 kV. The synchronization controller provides timing accuracy better than 1 μs, enabling simultaneous triggering of ignition and data acquisition. The dynamic data acquisition system is equipped with piezoelectric pressure sensors (range: 0–10 MPa) and photoelectric flame sensors (response time ≤ 1 μs), achieving a maximum sampling rate of 1 MHz per channel. It simultaneously collects explosion overpressure and flame signals.

### 2.2 Experimental Materials and Methods

High-purity methane, CO_2_, and N_2_ used in the experiment all had volume fractions of ≥99.99%, while HFC-227ea had a volume fraction of ≥99.9%, meeting industrial high-purity gas standards. All experiments adopted BOPP films with a fixed thickness of 75 μm. The dynamic rupture pressure under explosion loading was precalibrated using pressure sensors arranged on both sides of the film, resulting in a measured average value of 265 ± 5 kPa. This rupture pressure ensures that the BOPP film breaks during the stable propagation stage of methane deflagration, which aligns with the design intent of the nonpremixed suppression experiments. The compressed air used for gas mixing was dehydrated and dried using a molecular sieve to prevent interference from moisture and impurities on the experimental results. All experiments were conducted under standard ambient conditions at room temperature (25 ± 2 °C) and atmospheric pressure (101.325 kPa). The peak explosion overpressure and average flame propagation speed served as the core evaluation indicators, with primary monitoring focused on overpressure evolution and flame propagation characteristics within the suppression zone. The specific experimental procedure is as follows:

(1) A BOPP film of the specified thickness was installed to separate the pipeline sections, followed by system air tightness inspection and equipment trigger timing calibration.
(2) The explosion zone and suppression zone were individually evacuated. A 9.5% stoichiometric methane–air premixed gas and the explosion suppression medium at the designated concentration were prepared using the partial pressure method, and the mixture was allowed to stand for 5 min to ensure thorough gas mixing.
(3) Ignition was triggered by the synchronization controller, simultaneously initiating data acquisition to capture the complete explosion process.
(4) Each experimental condition was repeated three times, with the data relative deviation controlled within 5%. After each test, the pipeline was purged with dry air to eliminate residual gas interference, ensuring the experimental repeatability and safety.
(5) The flame arrival time at each measuring point is defined as the moment when the output signal of the photoelectric flame sensor first rises to 10% of its peak value, which is a widely adopted criterion in deflagration flame propagation experiments. The average flame propagation speed is calculated using the time-of-flight method. For each pair of adjacent flame sensors in the stable propagation section of the suppression zone (*x* = 8.5–11.5 m), the local flame speed is obtained by dividing the spacing between two adjacent measuring points (Δ*x* = 3.0 m) by the time difference (Δ*t*) of the flame front passing through the two points. The overall average flame propagation speed under each working condition is the arithmetic mean of the local speeds of all adjacent sensor pairs in the stable propagation section. The film rupture position only serves as the starting boundary of the suppression zone and is not involved in the speed difference calculation, so it has no influence on the accuracy of the average flame speed result.

### 2.3 Experimental Design

A 9.5% stoichiometric methane–air premixed gas was fixed in the explosion zone, and all tests were conducted under standard ambient temperature and pressure conditions. Three categories of experiments were designed—blank control, single-component suppression, and mixed-system synergistic suppression—to systematically quantify the suppression efficiency of different explosion suppression systems. The specific experimental conditions are as follows:

(1) Single-component suppression experiments: To establish the concentration–suppression efficiency relationship for individual suppressants, a blank control group (suppressant volume fraction of 0%) was included. For CO_2_ and N_2_, concentration gradients were set within the range of 32–40% (in increments of 2%), corresponding to the critical inerting range of the methane–air system. For HFC-227ea, the concentration range was 2–8% (also in increments of 2%). Detailed settings are provided in Table 1.
(2) Mixed suppression experiments: To investigate the synergistic explosion suppression effect of inert gases combined with HFC-227ea, the total volume fraction of the suppressant was fixed at 40%. The inert gas was progressively replaced by HFC-227ea, resulting in two types of mixed systems with four composition gradients, thereby enabling the coupling of regulation of physical dilution and chemical suppression. Detailed experimental conditions are provided in Table 2.

**Table 1:** Volume Fraction Settings for Single-Component Suppression Experiments

gas type	volume fraction (%)
CO_2_	0, 32, 34, 36, 38, 40
N_2_	0, 32, 34, 36, 38, 40
HFC-227ea	0, 2, 4, 6, 8

**Table 2:** Experimental System of Mixed Explosion Suppression and Setting of the Concentration Ratio

case no.	experimental system	volume ratio of mixed system (inert gas/HFC-227ea)
1-1	CO_2_/HFC-227ea	38%/2%
1-2		36%/4%
1-3	mixture system	34%/6%
1-4		32%/8%
2-1	N_2_/HFC-227ea	38%/2%
2-2		36%/4%
2-3	mixture system	34%/6%
2-4		32%/8%

All experimental conditions were tested in triplicate. Quantitative results (peak overpressure and average flame speed) are reported as the mean ± standard deviation of three replicates, with a relative standard deviation within 5%. The standard deviations are visualized as error bars in all quantitative comparison figures. Representative pressure–time curves are presented for clarity. The explosion suppression efficiency was quantitatively characterized using the reduction rates of the peak explosion overpressure and average flame propagation speed as the core indicators.

## 3 Results and Discussion

### 3.1 Explosion Suppression Effect of Single-Component Gas

#### 3.1.1 Explosion Suppression Characteristics of Inert Gas

The dynamic pressure–time evolution characteristics at measurement point P3 (downstream of the film) under various CO_2_ volume fractions are presented in Figure 2a. Upon film rupture, an instantaneous pressure jump accompanied by significant pressure oscillations was observed across all test conditions. In the blank control group (0% CO_2_), the peak overpressure occurred at the third oscillation peak; following the addition of CO_2_, the peak advanced to the second peak, and the pressure magnitude at each peak systematically decreased with increasing CO_2_ concentration. This phenomenon reflects the dynamic suppression mechanism of CO_2_: the turbulence induced by film rupture causes an initial pressure surge, while increasing CO_2_ concentration alters the density and constant-volume heat capacity of the gas mixture, thereby accelerating turbulent energy dissipation and heat loss and consequently weakening the superposition and intensification of pressure waves.^21^

**Figure 2:**
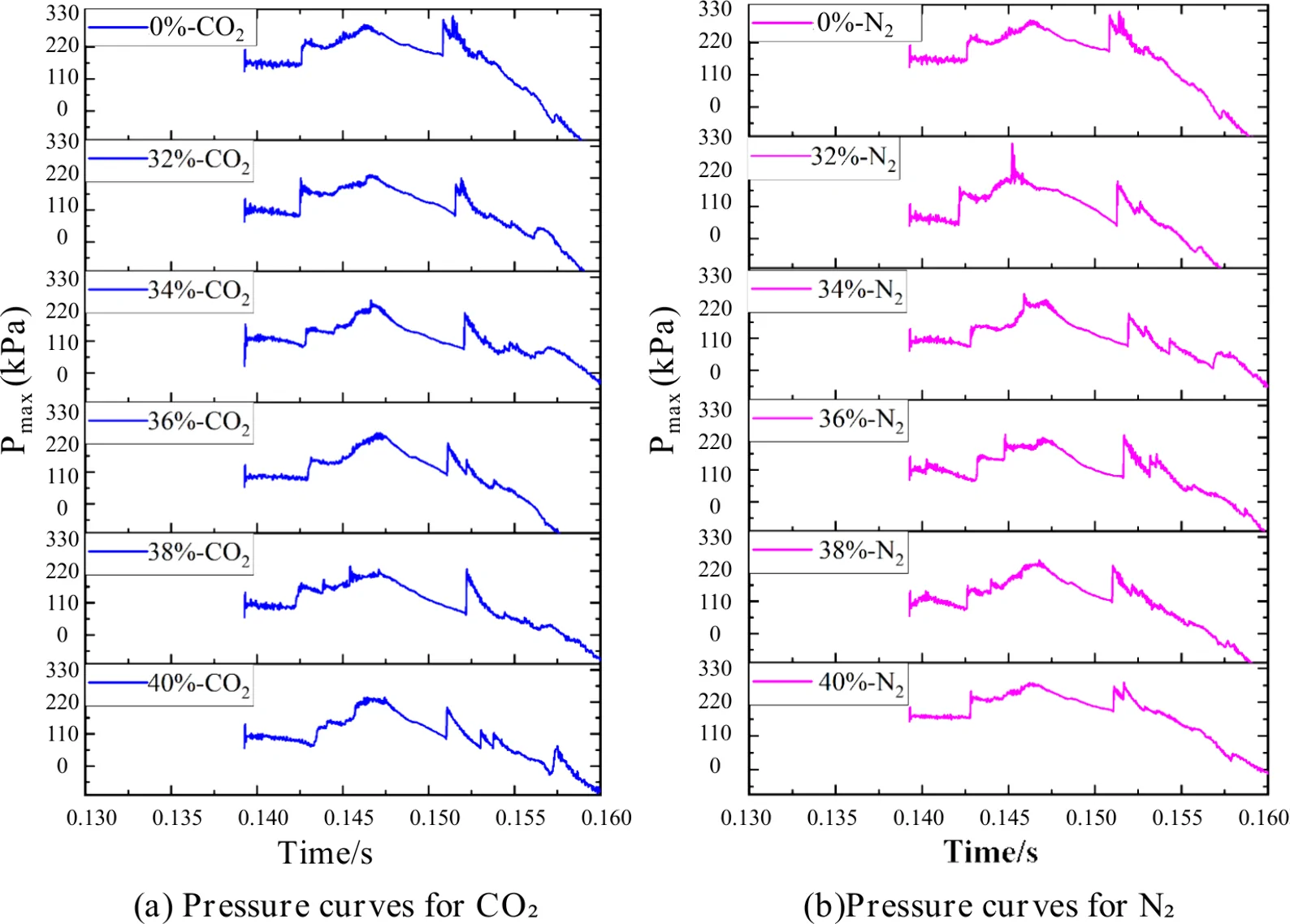
Time–history curve of pressure at point P3 behind the membrane under different volume fractions of CO_2_/N_2_.

Further analysis reveals that when the CO_2_ volume fraction increases to 38–40%, the overpressure peak appears significantly earlier than in the 32–36% range. This behavior can be interpreted through variation in the Damköhler number (*Da*): as CO_2_ concentration rises, the methane oxidation reaction rate decreases markedly, shifting the system from the chemical reaction-controlled regime (*Da* > 1) to the turbulent mixing-controlled regime (*Da* < 1).^22^ At a CO_2_ concentration of 38%, the dilution and thermal cooling effects of CO_2_ gradually approach their limiting efficacy. Because CO_2_ suppresses explosions primarily through physical thermodynamic dilution rather than efficient radical scavenging, further increasing the concentration yields diminishing marginal returns. When the CO_2_ concentration rises to 40%, the peak overpressure is only reduced by an additional 0.8%, indicating a distinct saturation effect in suppression. At the microscopic level, CO_2_ mainly relies on its high molar heat capacity to lower the flame temperature and reduce the oxygen partial pressure, while its direct chemical scavenging effect on free radicals is very weak. Therefore, the observed saturation of the suppression efficiency at high CO_2_ concentrations is attributed to the diminishing returns of physical dilution and thermal cooling.^23^

Figure 2b presents the pressure–time dynamic curves at measurement point P3 downstream of the film under varying N_2_ volume fractions, with the blank control group exhibiting a peak overpressure of 329.6 kPa. The results show that, at N_2_ volume fractions of 32%, 34%, 36%, 38%, and 40%, the peak explosion overpressures were 308.4, 289.0, 261.5, 256.3, and 248.7 kPa, respectively, corresponding to reductions of 6.3%, 12.2%, 20.5%, 22.3%, and 24.5% compared to the blank control. In the N_2_ concentration range of 32–36%, the peak overpressure and its reduction rate exhibited substantial changes, indicating that the suppression effect of N_2_ had not yet reached saturation within this range. Notably, the incremental enhancement in the suppression efficiency was greater for N_2_ than for CO_2_ in this interval. The primary reason is that CO_2_ possesses both physical dilution and weak chemical inhibition effects,^24^ approaching saturation suppression at lower concentrations, whereas N_2_, which relies solely on physical dilution and thermal cooling, retains a larger margin for efficiency improvement. As the N_2_ volume fraction increased to 38–40%, the suppression effect tended to stabilize. This stabilization is attributed to the vortex zone formed after film rupture promoting thorough mixing of N_2_ with the flame front, allowing the dilution-based suppression effect of N_2_ to be fully realized at the existing concentration; further enhancement would require an increase in concentration.^25^

As shown in Figure 3, the suppression effects of CO_2_ and N_2_ on the average flame propagation velocity of methane explosion exhibit a consistent macroscopic trend: the average flame velocity decreases continuously with increasing gas volume fraction, while the rate of reduction gradually diminishes (the flame propagation velocity under the baseline condition without suppression is 341.9 m/s). However, notable differences exist in both the suppression efficiency and underlying mechanisms between the two gases.

**Figure 3:**
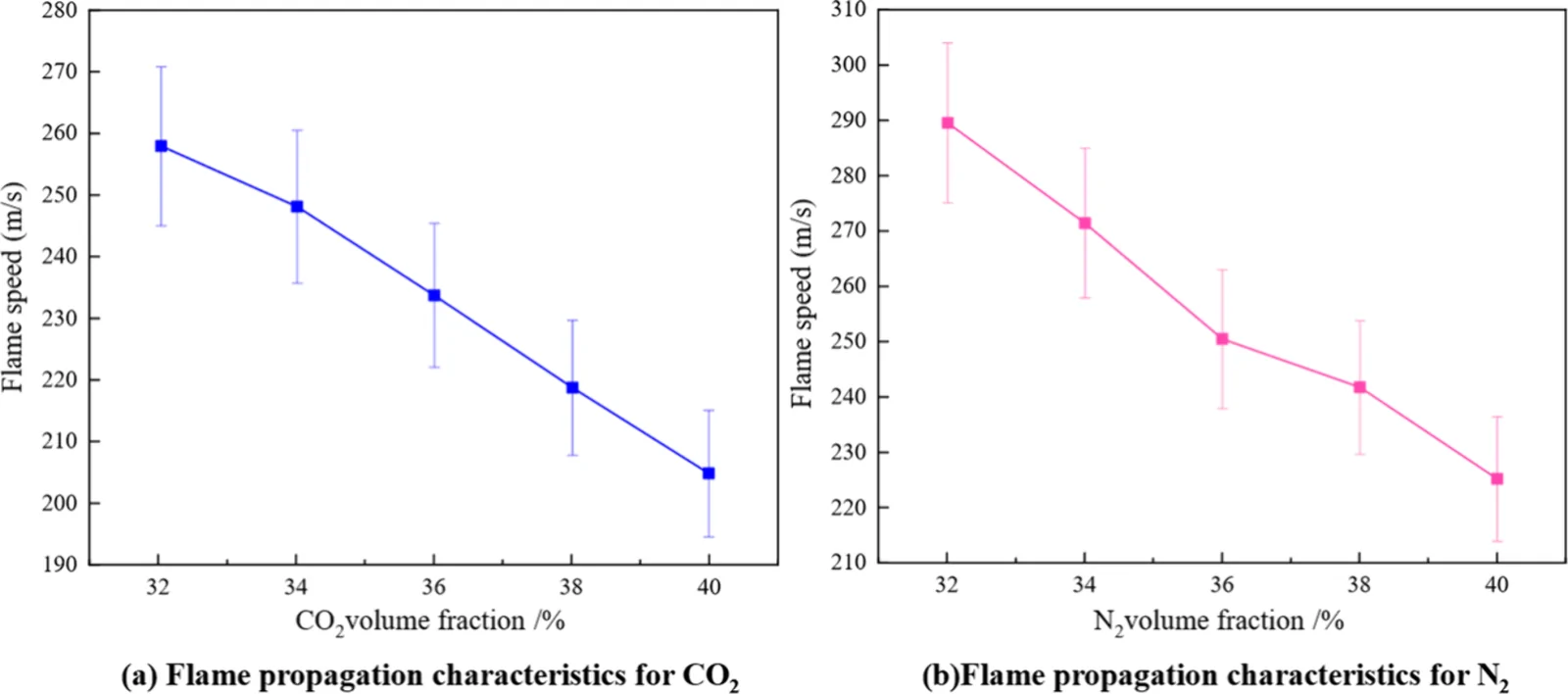
Variation curves of the average propagation velocity and decline rate of methane explosion flame under the action of different volume fractions of inert gas.

When the CO_2_ volume fraction ranges from 32% to 40%, the average flame propagation velocity decreases from 257.9 to 204.8 m/s, corresponding to reductions of 24.6–40.1% relative to the baseline condition. Under the same volume fractions, the average flame propagation velocities for N_2_ range from 289.3 to 225.6 m/s with reduction rates of only 15.4–34.0%, indicating significantly lower suppression efficiency compared to CO_2_. Furthermore, the inhibitory effect of CO_2_ on the average flame velocity is notably stronger than its regulation of the peak overpressure. This is mainly driven by physical effects: the intense turbulence generated upon BOPP film rupture temporarily increases the flame velocity but also accelerates the mixing of CO_2_ with the flame front, enhancing the dilution and cooling effects. According to previous kinetic studies, CO_2_ has a very weak chemical effect on free radicals such as H^•^ and OH^•^, and its suppression is dominated by the high molar heat capacity and oxygen dilution effect, which jointly reduce the reaction rate and slow down flame propagation.^23^ These effects, combined with the reverse retardation of the flame front by reflected pressure waves at the pipeline end, collectively strengthen the suppression of flame propagation.

The lower suppression efficiency of N_2_ is primarily attributed to the limitations of its inhibition mechanism. Although N_2_ has a slightly higher specific heat at constant pressure than CO_2_, it lacks a strong radiation absorption capacity, resulting in lower volumetric heat absorption efficiency and thus limited physical cooling. Moreover, CO_2_ can participate in chemical inhibition by directly scavenging chain-carrying radicals to interrupt the chain reaction, whereas N_2_ acts solely through physical means such as oxygen dilution and heat absorption without any chemical inhibition capability. Consequently, the overall explosion suppression efficiency of N_2_ is substantially inferior to that of CO_2_.^26,27^

#### 3.1.2 Explosion Suppression Characteristics of HFC-227ea

The evolution of the pressure–time history at measurement point P3 and the average flame velocity downstream of the film under the action of HFC-227ea (HFC-227ea) at volume fractions ranging from 2% to 8% are shown in Figure 4. The experimental results demonstrate that the explosion suppression efficiency of HFC-227ea is significantly superior to that of inert gases such as CO_2_, with a volume fraction of 8% achieving highly efficient explosion suppression. Its core advantage lies in the deep synergy between physical and chemical suppression mechanisms, yielding overall suppression effects on overpressure and flame propagation that far exceed those of single inert gases.^28^

**Figure 4:**
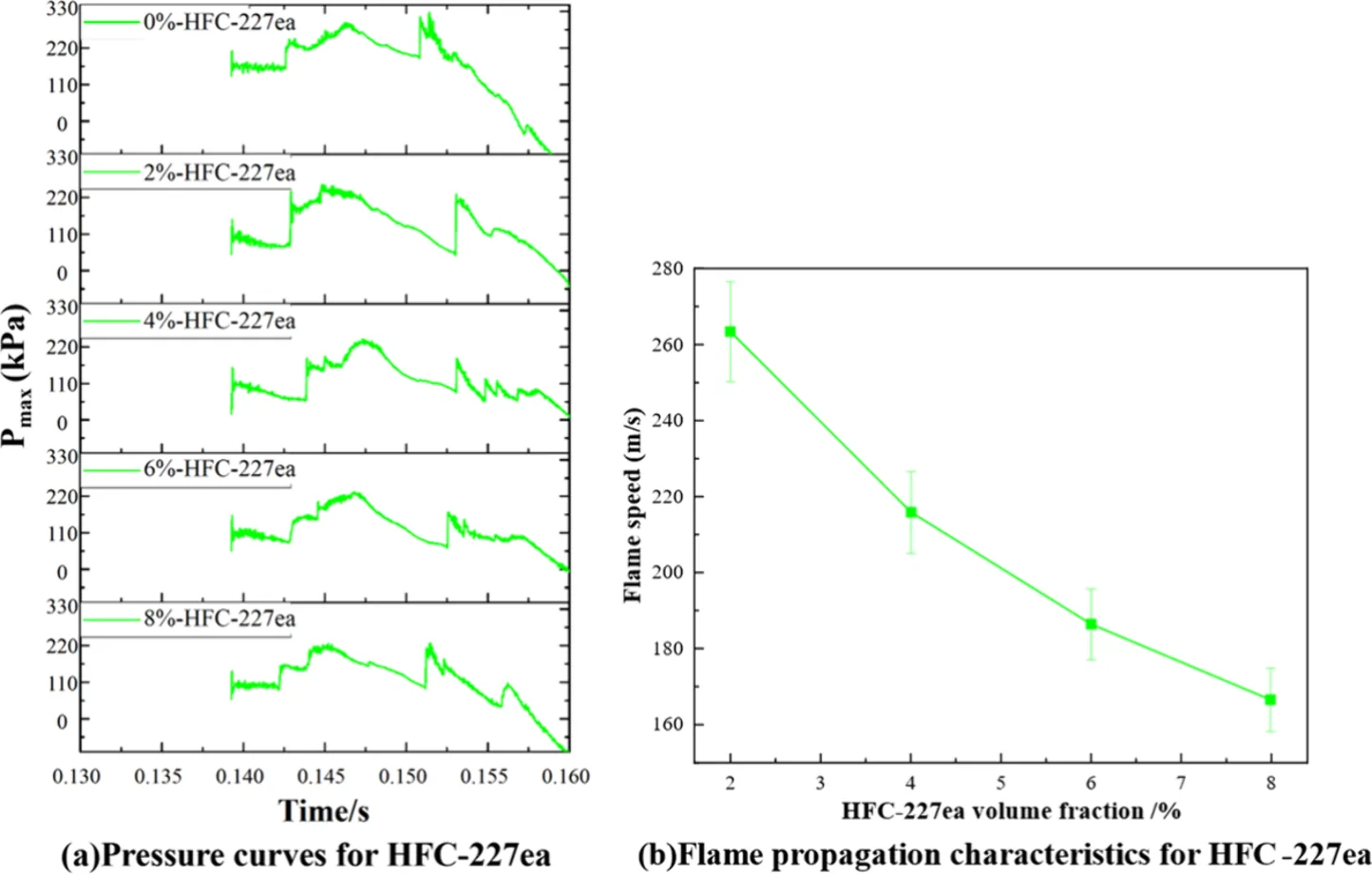
Variation curve of methane explosion characteristics under the action of different volume fractions of HFC-227ea.

Under the baseline condition without suppressant, the flame propagation velocity is 341.9 m/s. As the HFC-227ea volume fraction increases from 2% to 8%, the average flame propagation velocity continuously decreases from 263.4 to 166.3 m/s, corresponding to reduction rates increasing linearly from 23.0% to 51.4% relative to the baseline condition. The explosion overpressure peak appears at the second peak across all HFC-227ea test conditions, confirming its rapid suppression characteristics. The arrival time of the overpressure peak exhibits a nonlinear dependence on concentration: at 2% concentration, the vortex generated by film rupture promotes rapid mixing of the suppressant with the explosive gas, leading to earlier suppression onset; at 4% concentration, the delay in peak timing arises from the competition between the enhanced chemical inhibition and the physical dilution/turbulent mixing process. The chemical suppression delays the energy release; however, the localized turbulence induced by the film rupture still requires a finite time to fully mix and disperse the suppressant, leading to a slight delay in the superposition of pressure waves; at higher concentrations of 6–8%, the abundant suppressant continues to exert its effect during the pressure wave reflection stage, advancing the peak time again by reducing the amplitude of late-stage pressure oscillations.

The suppression effect of HFC-227ea on the flame propagation velocity is notably stronger than its effect on overpressure regulation. This disparity arises from the multifield coupled synergistic explosion suppression mechanism. Although the intense turbulence generated upon film rupture transiently increases the flame speed, the accompanying vortices promote local enrichment of HFC-227ea at the flame front, forming a uniform inhibitor distribution zone. Based on existing combustion kinetic studies, the suppression effect of HFC-227ea comes from the coupling of physical and chemical mechanisms:^29,30^

(1) Chemical inhibition: HFC-227ea decomposes rapidly at high flame temperatures to produce fluorine-containing active species (such as F^•^ and CF_*x*_^•^), which quench key chain carriers (H^•^ and OH^•^) through chain termination reactions and interrupt the explosion chain reaction.
(2) Physical cooling: The phase change and pyrolysis process of HFC-227ea absorb a large amount of heat, reducing the flame zone temperature and slowing the reaction rate according to the Arrhenius law.

### 3.2 Explosion Suppression Effect of Mixed Systems

#### 3.2.1 Analysis of the Explosion Suppression Performance of Mixed Systems

Figure 5 presents the pressure–time history curves at measurement point P3 downstream of the film under the action of CO_2_/HFC-227ea mixed systems with different proportions, with the total volume fraction of the suppressant fixed at 40%. The proportion gradients examined are 38%/2%, 36%/4%, 34%/6%, and 32%/8% (CO_2_/HFC-227ea). Compared with the baseline condition and the pure CO_2_ control group, the overpressure peaks of all mixed systems are reduced to varying degrees, and the peak position shifts from the third oscillation peak observed in the baseline condition to the second peak. This indicates that the mixed system rapidly exerts its inhibitory effect during the turbulent mixing stage following film rupture, thereby suppressing the superimposed growth of explosion pressure.

**Figure 5:**
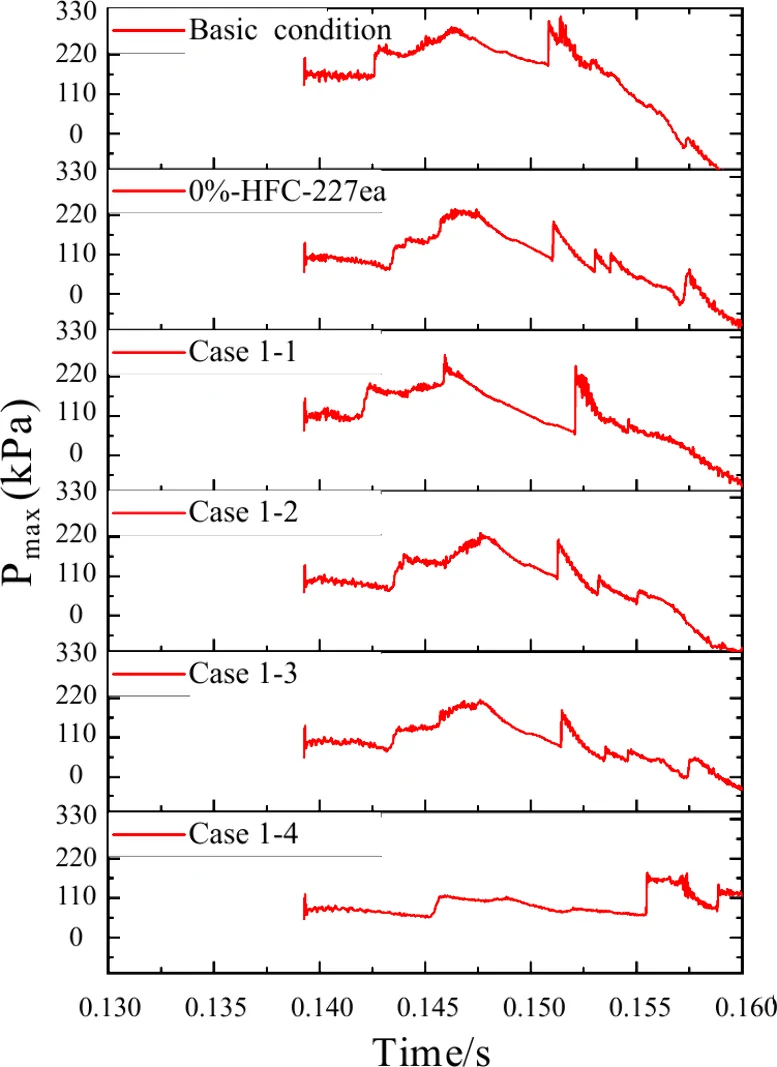
Curves of pressure at point P3 under the action of CO_2_/HFC-227ea mixed systems with different proportions.

The explosion suppression mechanisms of CO_2_ and HFC-227ea exhibit a significant complementarity. CO_2_ primarily acts through physical inhibition, achieving thermal dilution and cooling via its high molar heat capacity while also reducing the oxygen partial pressure to dilute combustible components with only weak chemical inhibition. In contrast, the core advantage of HFC-227ea lies in its highly efficient chemical inhibition, wherein fluorine-containing free radicals generated by pyrolysis rapidly quench the key active intermediates involved in the explosive chain reaction. When combined, these two suppressants form complementary advantages: CO_2_ compensates for the limited physical heat absorption and dilution capacity of HFC-227ea, while HFC-227ea significantly enhances the chemical inhibition efficiency of the system, collectively achieving an overall improvement in the explosion suppression performance. Analysis of the overpressure peak arrival time reveals that, except for the 38%/2% mixture condition, where the peak time remains essentially unchanged or slightly earlier than that of the baseline condition, the peak arrival times for the other mixed systems are notably delayed compared to the baseline, with the delay amplitude progressively increasing with the proportion of HFC-227ea. Among these, the 32%/8% proportion exhibits the most pronounced time-lag effect. It is important to note that this delay is not due to the decomposition kinetics of HFC-227ea (which decomposes extremely rapidly under flame conditions). Instead, it is attributed to the strong chemical chain termination effect of HFC-227ea, which effectively interrupts the chain reactions and reduces the overall heat-release rate. This decrease in the energy release rate delays the accumulation and superposition of pressure waves, and this condition also yields the lowest overpressure peak and pressure oscillation amplitude among all tested conditions. This indicates that, with an increased proportion of HFC-227ea, the mixed system significantly retards the growth rate of explosion pressure through sustained chemical inhibition, substantially weakening the destructiveness of the explosion and markedly enhancing the overall explosion suppression efficiency.

Figure 6 presents the pressure–time history curves at measurement point P3 downstream of the film under the action of N_2_/HFC-227ea mixed systems with different proportions, with the total volume fraction of the suppressant fixed at 40%. The proportion gradients examined are 38%/2%, 36%/4%, 34%/6%, and 32%/8% (N_2_/HFC-227ea). The peak overpressure under the baseline condition is 329.6 kPa. According to the experimental data, at mixture ratios of 38%/2%, 36%/4%, 34%/6%, and 32%/8%, the corresponding explosion overpressure peaks are 241.2, 236.4, 222.5, and 218.9 kPa, respectively, representing reductions of 26.7%, 28.1%, 32.4%, and 33.6% relative to the baseline condition. Compared with the pure N_2_ condition at a volume fraction of 40%, the peak overpressures are reduced by 3.0%, 4.9%, 10.5%, and 12.1%, respectively.

**Figure 6:**
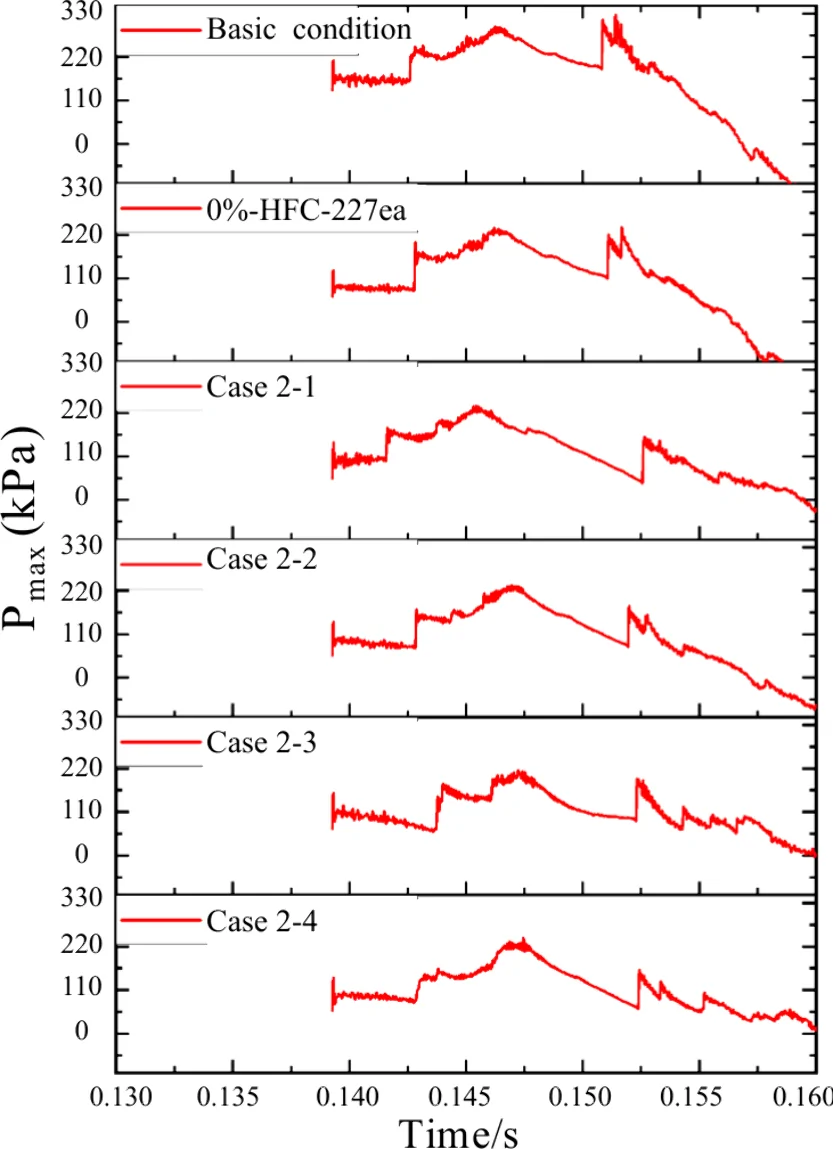
Variation of the peak overpressure and reduction rate at point P3 of N_2_/HFC-227ea mixed systems with different proportions.

The experimental results indicate that the addition of HFC-227ea to the pure N_2_ system effectively enhances methane explosion suppression, with the suppression effect continuously increasing as the volume fraction of HFC-227ea in the mixed system increases. Comparison with the experimental results of the CO_2_/HFC-227ea mixed system reveals that when the HFC-227ea volume fraction is in the range of 2–4%, the N_2_-based mixed system exhibits a more significant peak overpressure suppression effect. However, when the HFC-227ea volume fraction increases to the range of 6–8%, the CO_2_-based mixed system demonstrates a superior suppression performance in terms of peak overpressure.

In summary, for both types of inert gases, the explosion suppression efficiency of the mixed systems improves markedly with an increasing HFC-227ea volume fraction. For the CO_2_/HFC-227ea system, at the 32%/8% ratio, the peak overpressure is reduced by 35.3% compared to the baseline condition, representing a 9.5 percentage point increase over the 40% pure CO_2_ condition. The flame propagation speed is reduced by 62.9% relative to the baseline condition, corresponding to a 22.8 percentage-point improvement over the pure CO_2_ condition. For the N_2_/HFC-227ea system, at the 32%/8% ratio, the peak overpressure and flame propagation speed are reduced by 33.6% and 59.5%, respectively, compared with the baseline condition and by 12.1% and 24.8%, respectively, relative to the 40% pure N_2_ condition. These results demonstrate that the addition of a small amount of HFC-227ea can significantly reduce the required inert gas concentration while maintaining an effective explosion suppression. Although a detailed cost-benefit analysis is beyond the scope of this experimental study, this reduction in inert gas consumption suggests the potential to reduce the operational costs associated with large-scale gas storage and transportation in practical engineering applications.

#### 3.2.2 Analysis of the Synergistic Effect of Two Explosion Suppressants

Figure 7 illustrates the pressure–time history curves at measurement point P3 downstream of the film under the action of CO_2_, HFC-227ea single-component systems, and their mixed systems at various proportions, with the total volume fraction of the suppressant fixed at 40%. Parts a–d of Figure 7 correspond to gradient conditions with HFC-227ea volume fractions of 2%, 4%, 6%, and 8%, respectively. The peak overpressure under the baseline condition (without suppressant) is 329.6 kPa.

**Figure 7:**
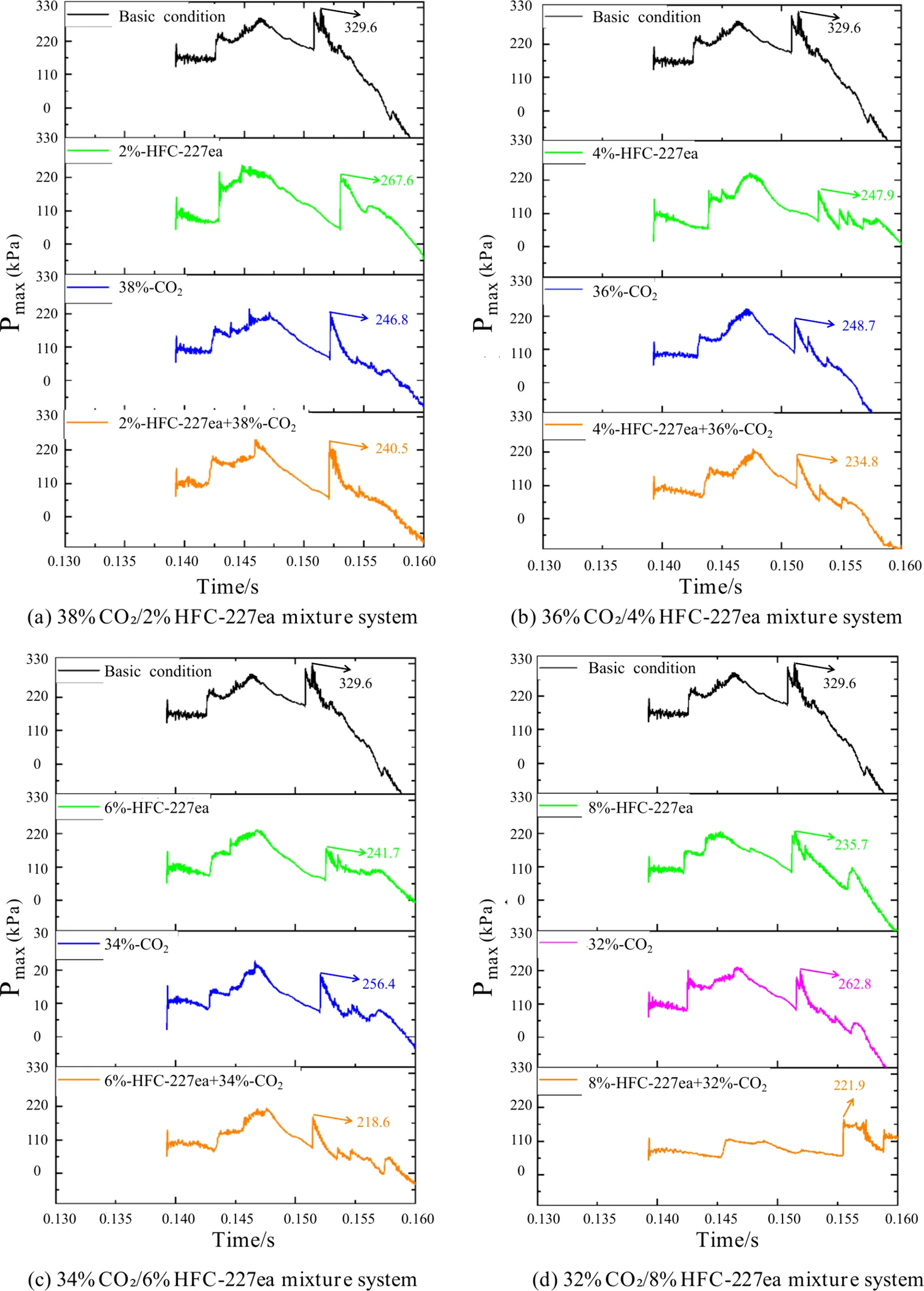
Pressure–time history curves at point P3 of CO_2_/HFC-227ea mixed systems with different ratios.

The experimental results show that when 2% HFC-227ea and 38% CO_2_ are applied individually, the peak overpressure reduction rates are 18.7% and 25.8%, respectively. When combined as the 38%/2% mixed system, the reduction rate increases to 26.9%. For 4% HFC-227ea and 36% CO_2_ applied individually, the reduction rates are 24.7% and 24.4%, respectively, while the 36%/4% mixed system achieve a reduction rate of 28.6%. For 6% HFC-227ea and 34% CO_2_ applied individually, the reduction rates are 26.5% and 22.1%, respectively, and the 34%/6% mixed system yields a reduction rate of 33.6%. For 8% HFC-227ea and 32% CO_2_ applied individually, the reduction rates are 28.4% and 20.1%, respectively, while the 32%/8% mixed system achieves a reduction rate of 35.3%.

From the evolution characteristics of the pressure–time history curves, it is evident that the peak overpressure reduction rate of every mixed system exceeds that of the corresponding single-component systems under the same gradient conditions, with the synergistic suppression effect gradually strengthening as the proportion of HFC-227ea increases. Additionally, when CO_2_ and HFC-227ea are combined, the arrival time of the overpressure peak is delayed to varying degrees compared with that of either single-component system alone, and the magnitude of this delay increases with the proportion of HFC-227ea. This indicates that the mixed system significantly retards the release rate of explosion energy, resulting in a marked improvement in methane explosion suppression compared with single-component systems.

Figure 8 shows the pressure–time history curves at measurement point P3 downstream of the film under the action of N_2_, HFC-227ea single-component systems, and their mixed systems at various proportions, with the total volume fraction of the suppressant fixed at 40%. Parts a–d of Figure 8 correspond to gradient conditions with HFC-227ea volume fractions of 2%, 4%, 6%, and 8%, respectively. The peak overpressure under the baseline condition (without suppressant) is 329.6 kPa.

**Figure 8:**
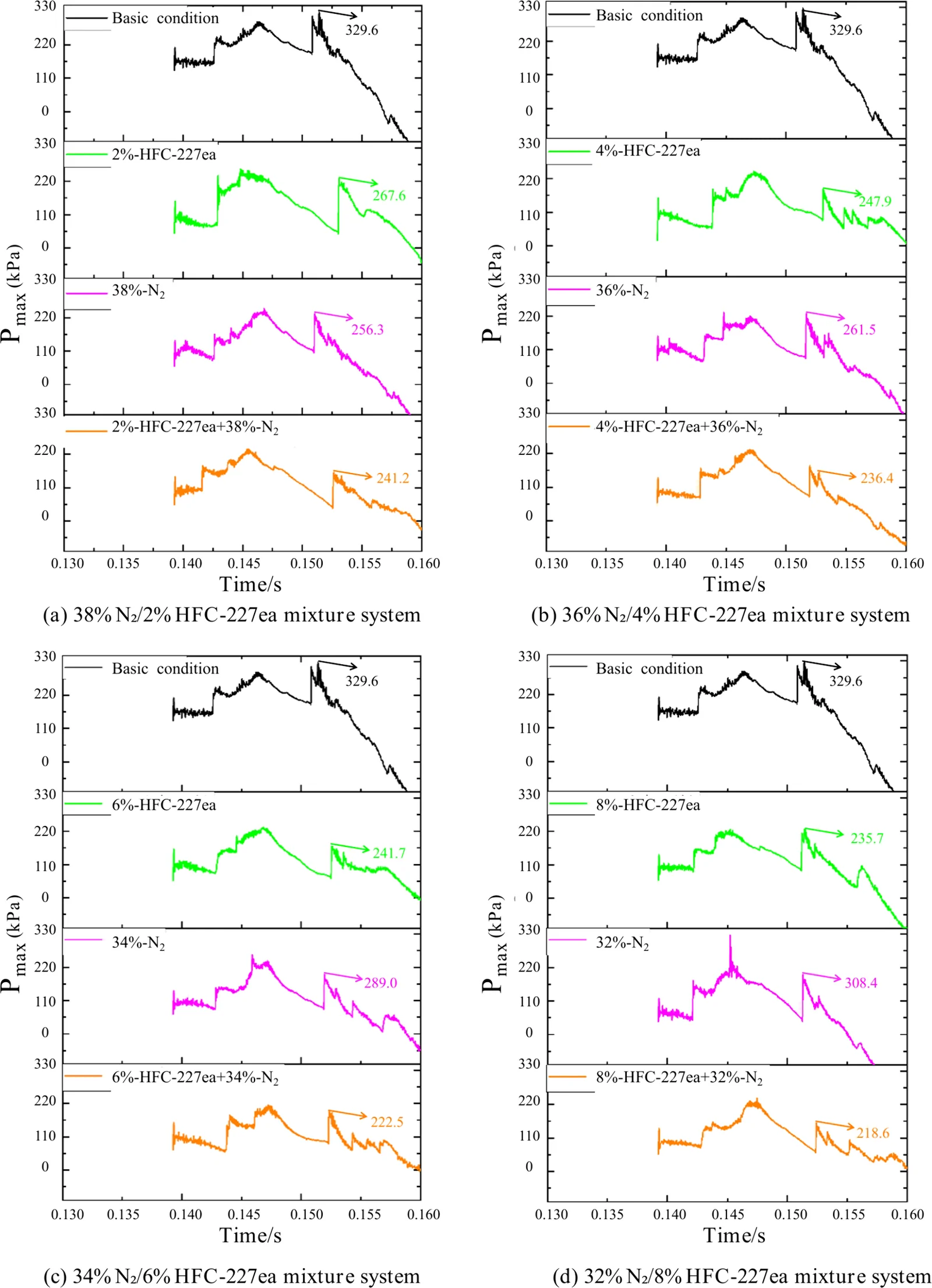
Pressure–time history curves at point P3 of N_2_/HFC-227ea mixed systems with different ratios.

The experimental results show that when 2% HFC-227ea and 38% N_2_ are applied individually, the peak overpressure reduction rates are 18.7% and 22.1%, respectively. When combined as the 38%/2% mixed system, the reduction rate increases to 26.7%. For 4% HFC-227ea and 36% N_2_ applied individually, the reduction rates are 24.7% and 20.5%, respectively, while the 36%/4% mixed system achieves a reduction rate of 28.1%. For 6% HFC-227ea and 34% N_2_ applied individually, the reduction rates are 26.5% and 12.2%, respectively, and the 34%/6% mixed system yields a reduction rate of 32.4%. For 8% HFC-227ea and 32% N_2_ applied individually, the reduction rates are 28.4% and 6.3%, respectively, while the 32%/8% mixed system achieves a reduction rate of 33.6%.

From the evolution characteristics of the pressure–time history curves, it is evident that the peak overpressure reduction rate of every mixed system is significantly higher than that of the corresponding single-component systems under the same gradient conditions, with the synergistic suppression effect increasing nonlinearly as the proportion of HFC-227ea increases. Additionally, when N_2_ is combined with HFC-227ea, the arrival time of the overpressure peak is notably delayed compared with that of either single-component system alone, and the magnitude of this delay continues to increase with the proportion of HFC-227ea. This indicates that the mixed system effectively retards the energy release process of the explosion, resulting in a substantial improvement in methane explosion suppression compared with single-component systems. Notably, under conditions with low N_2_ concentration, the addition of HFC-227ea achieves a leap-forward enhancement in the explosion suppression efficiency.

Comparing the explosion suppression effects of the mixed systems with those of single components, we find that, although the suppression performance of the mixed gases surpasses that of a single inert gas, no significant synergistic enhancement effect is observed. Taking the 34% CO_2_/6% HFC-227ea mixture as an example, its peak overpressure reduction rate (33.6%) is slightly higher than the weighted average of the individual components—22.1% for 34% CO_2_ and 26.5% for 6% HFC-227ea—but does not exhibit a pronounced “1 + 1 > 2” effect relative to the theoretical superposition of the single-component suppression efficiencies.

It is inferred that this phenomenon is related to the film-breaking effect. In this study, the dynamic rupture pressure of the 75-μm-thick BOPP film under explosion loading was precalibrated to be 265 ± 5 kPa, which ensures that the film ruptures during the stable deflagration stage. However, direct characterization of the rupture dynamics and downstream turbulence intensity was not performed. The inferred mechanism is as follows: First, the BOPP film physically separates the premixed methane gas from the suppressant, so the suppressant cannot act in the initial stage of explosion. Second, the film ruptures only when the flame front arrives, by which time the explosion in the upstream pipeline has accumulated considerable intensity through repeated pressure-wave superposition. More importantly, the rupture process itself generates strong compression waves, causing an instantaneous overpressure surge downstream of the film, and the accompanying turbulence accelerates flame propagation to a certain extent.^31^ These factors jointly weaken the full expression of the synergistic effect of the mixed suppressant, making the theoretical synergistic suppression mechanism not fully realized under the current nonpremixed experimental conditions.

#### 3.2.3 Analysis of the Explosion Suppression Mechanism of Mixed Systems

On the basis of existing literature and known combustion kinetics,^29,30,32^ we hypothesize the following synergistic explosion suppression mechanism for the mixed system. N_2_ and HFC-227ea form a deep complementarity of physical and chemical actions along the explosion suppression pathway, enabling full-dimensional intervention in the methane explosion chain reaction from the macroscopic flow field regulation to microscopic free-radical quenching. While this study does not involve direct radical species measurements, the following mechanistic pathway is inferred from the established chemical principles. The synergistic explosion suppression mechanism can be systematically elucidated by combining the reaction pathways and action mechanisms illustrated in Figure 9.

**Figure 9:**
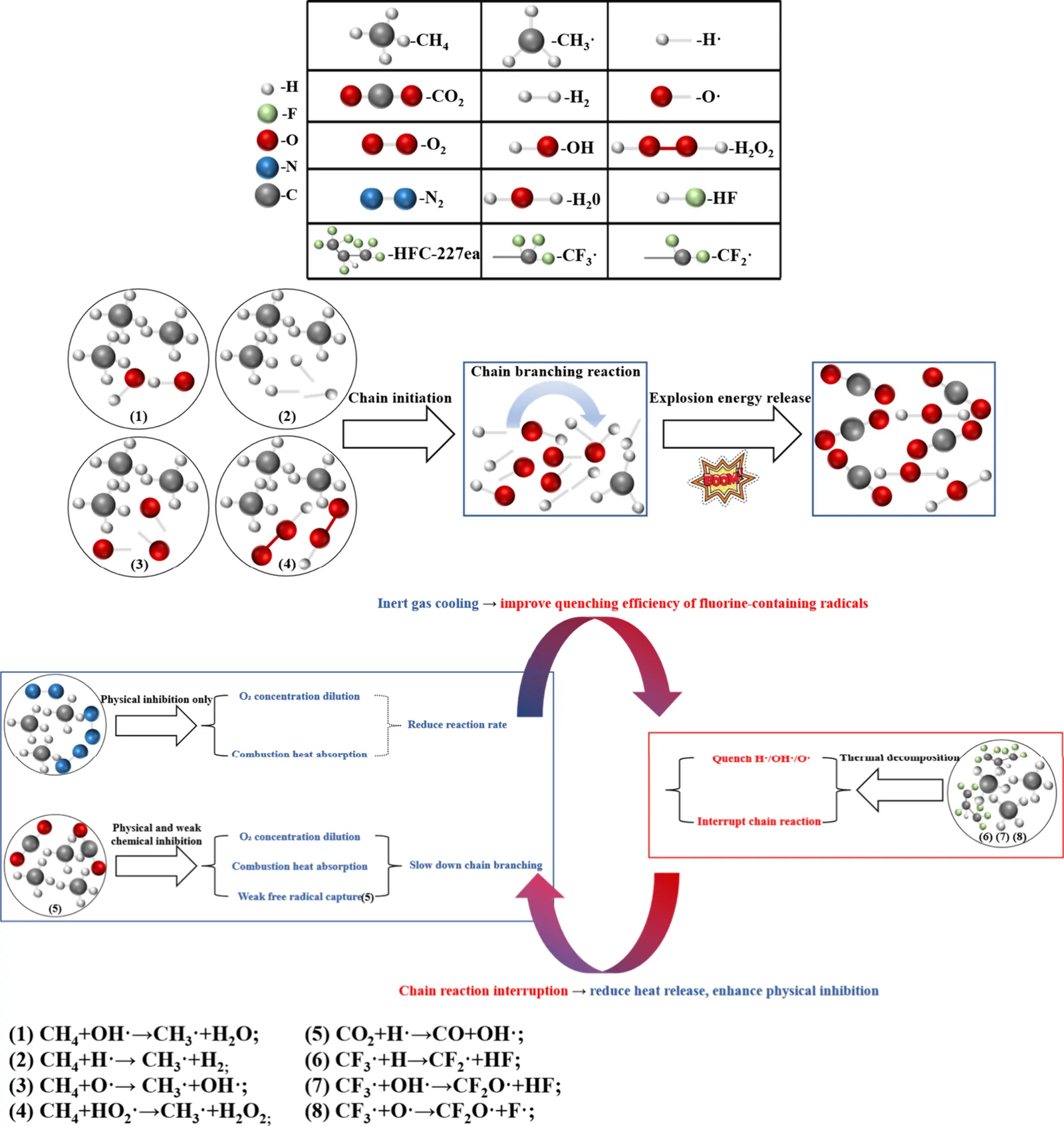
Schematic diagram of the hypothesized suppression mechanism of the inert gas/HFC-227ea mixed system (inferred from existing literature).

Pure N_2_ functions solely through physical mechanisms, namely, oxygen concentration dilution and combustion heat absorption, and is incapable of capturing or quenching key active free radicals such as H^•^, OH^•^, and O^•^ that sustain the methane explosion chain reaction. Its lack of chemical inhibition capability is the core reason why its explosion suppression efficiency remains limited even at high concentrations. The introduction of HFC-227ea adds an efficient chemical inhibition pathway to the system. Upon thermal decomposition in the high-temperature flame zone, HFC-227ea generates fluorine-containing active intermediates (e.g., CF_*x*_), which react with the core chain carriers of the explosive chain reaction through multistep elementary reactions, rapidly consuming free radicals, thereby interrupting the self-sustaining propagation of the chain reaction and fundamentally compensating for the suppression deficiencies of the pure N_2_ system. Simultaneously, the physical dilution and cooling effects of N_2_ and the chemical chain-breaking effect of HFC-227ea form a bidirectional synergy. N_2_ lowers the reaction zone temperature by reducing the oxygen partial pressure and absorbing combustion heat, which slows the chain-branching reaction rate of methane oxidation and provides a more favorable environment for the quenching reactions of fluorine-containing free radicals. Conversely, the interruption of the chain reaction by HFC-227ea reduces the system’s heat release rate, further reinforcing the physical suppression effect of N_2_. This establishes a closed-loop physical–chemical dual suppression mechanism, ultimately achieving strong suppression of explosion overpressure growth and flame propagation.

Compared with the CO_2_/HFC-227ea mixed system, the addition of HFC-227ea yields a more pronounced improvement in the explosion suppression efficiency for the N_2_-based system. The underlying reason is that CO_2_ inherently possesses both strong physical inhibition (due to its high molar heat capacity) and weak chemical inhibition capability (participating in free-radical reactions); thus, the addition of HFC-227ea merely enhances its existing chemical suppression capacity. In contrast, pure N_2_ lacks any chemical inhibition property; with the addition of HFC-227ea, a chemical suppression pathway is introduced where none existed before, rendering its marginal contribution to the overall explosion suppression efficiency more significant. Furthermore, although the film-breaking effect attenuates the synergistic performance of the system to some extent, the resulting strong turbulence within the pipeline promotes rapid and uniform mixing of N_2_ and HFC-227ea at the flame front, greatly increasing the probability of contact between fluorine-containing radicals and explosive chain carriers, thereby ensuring the full realization of HFC-227ea’s chemical inhibition. This also explains why the mixed system still achieves robust explosion suppression under spatially separated conditions.

It should be acknowledged that this study has several limitations:

(1) The experimental pipeline is 12 m in length and 90 mm in inner diameter, which is smaller than that of actual industrial pipelines and roadway systems. The scale effect may not fully reproduce the explosion dynamics in large-scale complex environments, but the wave evolution and flame acceleration laws obtained are consistent with the general mechanism, and the conclusions can provide a reference for practical engineering scenarios.
(2) The tested volume fraction range of inert gas (32–40%) is selected based on the conventional critical inerting interval, which cannot cover all concentration and initial pressure conditions in industrial applications.
(3) This study did not carry out direct quantitative characterization of BOPP film rupture dynamics and downstream turbulence intensity, and the influence mechanism of the film-breaking effect on synergistic suppression was inferred from macroscopic experimental phenomena. Subsequent work will use high-speed imaging and particle image velocimetry for further verification.
(4) No direct detection of free-radical species and reaction pathways was carried out in this work. The proposed microscopic suppression mechanism is inferred from existing combustion kinetic studies and will be further verified by Chemkin kinetic simulation and flame spectral diagnosis in follow-up research.

## 4 Conclusions

Based on the nonpremixed conditions representative of underground coal mine gas explosion suppression, this study employed a horizontal pipeline experimental system with a BOPP film separating the explosion and suppression zones to investigate the methane explosion suppression characteristics of CO_2_, N_2_, and HFC-227ea single-component and mixed systems. The main conclusions are as follows:

(1) In the concentration range of 32–40%, the suppression efficiencies of both CO_2_ and N_2_ increased with the concentration, reaching the optimal performance within the tested concentration range at 40% CO_2_, possessing dual physical and weak chemical inhibition, and significantly outperforming N_2_, which relies solely on physical effects. At 40%, CO_2_ reduced the peak overpressure by 25.8% and the flame propagation speed by 40.1%. HFC-227ea exhibited excellent performance in the low concentration range of 2–8%, reducing the flame propagation speed by 51.4% at 8%, far exceeding inert gases.
(2) With the total suppressant volume fraction fixed at 40%, the suppression performance of both mixed systems improved with an increasing HFC-227ea proportion. The 32% CO_2_/8% HFC-227ea mixture achieved optimal performance, reducing the peak overpressure by 35.3% and the flame propagation speed by 62.9%, representing improvements of 9.5% and 22.8% over those of pure CO_2_ at 40%. This optimal ratio reduces the required inert gas concentration while maintaining a high explosion suppression efficiency, thereby offering a potential economic benefit by lowering the costs associated with large-scale inert gas storage and transportation in practical applications.
(3) It is inferred that the film-breaking effect is a key factor influencing synergistic suppression under nonpremixed conditions. The compression wave and intense turbulence generated by BOPP film rupture interfere with the interaction timing and mixing efficiency of the suppressant and methane premixed gas. Consequently, no significant positive synergistic enhancement was observed at low HFC-227ea proportions; only when the HFC-227ea proportion exceeded 6% did the physical–chemical synergistic effect gradually emerge. This finding clarifies the core discrepancy between premixed experiments and nonpremixed underground conditions, providing theoretical guidance for structural optimization, installation positioning, and membrane rupture pressure design of gas explosion suppression devices.

This study clarifies the explosion suppression characteristics of inert gas/HFC-227ea mixed systems under nonpremixed conditions, providing experimental support for efficient gas explosion suppression technology in coal mines. Future research will focus on two directions: (1) constructing detailed reaction kinetic models using Chemkin to reveal the microscopic essence of synergistic suppression and (2) quantifying the quantitative response relationships between the membrane rupture pressure, turbulence intensity, and suppression efficiency to define critical rupture conditions for synergistic suppression.

## Data Availability

The data that support the findings of this study are available from the corresponding author upon reasonable request.

## ○

College of Safety and Emergency Management Engineering, Taiyuan University of Technology, Taiyuan, Shanxi, 030024, China
